# Genome‐wide association study and genomic prediction for intramuscular fat content in Suhuai pigs using imputed whole‐genome sequencing data

**DOI:** 10.1111/eva.13496

**Published:** 2022-10-24

**Authors:** Binbin Wang, Pinghua Li, Liming Hou, Wuduo Zhou, Wei Tao, Chenxi Liu, Kaiyue Liu, Peipei Niu, Zongping Zhang, Qiang Li, Guosheng Su, Ruihua Huang

**Affiliations:** ^1^ Key Laboratory in Nanjing for Evaluation and Utilization of Pigs Resources Ministry of Agriculture and Rural Areas of China, Institute of Swine Science, Nanjing Agricultural University Nanjing China; ^2^ Center for Quantitative Genetics and Genomics Aarhus University Aarhus Denmark; ^3^ Huaian Academy Nanjing Agricultural University China; ^4^ Huaiyin Xinhuai Pig Breeding Farm of Huaian City China

**Keywords:** genomic prediction, GWAS, imputed WGS data, intramuscular fat content, pigs

## Abstract

Integrating the single‐nucleotide polymorphisms (SNPs) significantly affecting target traits from imputed whole‐genome sequencing (iWGS) data into the genomic prediction (GP) model is an economic, efficient, and feasible strategy to improve prediction accuracy. The objective was to dissect the genetic architecture of intramuscular fat content (IFC) by genome wide association studies (GWAS) and to investigate the accuracy of GP based on pedigree‐based BLUP (PBLUP) model, genomic best linear unbiased prediction (GBLUP) models and Bayesian mixture (BayesMix) models under different strategies. A total of 482 Suhuai pigs were genotyped using an 80 K SNP chip. Furthermore, 30 key samples were selected for resequencing and were used as a reference panel to impute the 80 K chip data to the WGS dataset. The 80 K data and iWGS data were used to perform GWAS and test GP accuracies under different scenarios. GWAS results revealed that there were four major regions affecting IFC. Two important functional candidate genes were found in the two most significant regions, including protein kinase C epsilon (*PRKCE*) and myosin light chain 2 (*MYL2*). The results of the predictions showed that the PBLUP model had the lowest reliability (0.096 ± 0.032). The reliability (0.229 ± 0.035) was improved by replacing pedigree information with 80 K chip data. Compared with using 80 K SNPs alone, pruning iWGS SNPs with the R‐squared cutoff of linkage disequilibrium (0.55) led to a slight improvement (0.006), adding significant iWGS SNPs led to an improvement of reliability by 0.050 when using a one‐component GBLUP, a further increase of 0.033 when using a two‐component GBLUP model. For BayesMix models, compared with using 80 K SNPs alone, adding additional significant iWGS SNPs into one‐ or two‐component BayesMix models led to improvements of reliabilities for IFC by 0.040 and 0.089, respectively. Our results may facilitate further identification of causal genes for IFC and may be beneficial for the improvement of IFC in pig breeding programs.

## 1 INTRODUCTION

With the improvement of people's living standards, pork quality has attracted more and more attention. Intramuscular fat content (IFC) is an important determinant of pork quality affecting multiple other meat quality indicators, such as flavor, shearing force, and texture (Cho et al., 2015). Moreover, the appearance of pork, such as marbling and meat color, will strongly influence the purchase intention of consumers (Ngapo, 2017). It is generally accepted that a higher level of IFC has a positive influence on the sensory experience associated with eating (Wood et al., 2004). Thus, there is an increased requirement for genetic improvement of IFC in pig breeding programs. However, genetic improvement of IFC by a traditional breeding method is challenging because the trait can only be measured accurately after slaughter.

Animal breeding has been revolutionized by genomic prediction (GP). In recent years, genomic information has been widely applied in livestock breeding, which greatly increases selection accuracy, particularly for those traits with low heritability, difficult or expensive to measure (e.g. meat quality) (Jia & Jannink, 2012). In GP, the selection of candidate animals is based on the genomic estimated breeding value (GEBV) using genome‐wide dense single‐nucleotide polymorphisms (SNPs). Some popular genomic evaluation approaches, such as genomic best linear unbiased prediction (GBLUP) and Bayesian variable selection methods, have been widely used in practical genomic evaluation (Salek Ardestani et al., 2021).

Genomic prediction using SNP chip genotype data depends on linkage disequilibrium (LD) between causal genes and SNP markers. It is expected that the GP using whole‐genome sequence (WGS) data will increase prediction accuracy because WGS data included causal SNPs affecting target traits, and the prediction is much less dependent on LD between SNPs and causal SNPs (Meuwissen & Goddard, 2010; Song et al., 2019). Despite the cost of WGS is rapidly decreasing, resequencing a large number of individuals is still expensive. An efficient approach to increase the number of animals with WGS SNP is to impute genotypes of chips to WGS (Larmer et al., 2017). Imputation is the process of inferring missing genotypes, such as inferring unknown genotypes for animals genotyped at lower SNP density by using a reference set of animals genotyped at a higher SNP density. However, a previous study showed that the direct use of imputed WGS (iWGS) data for prediction did not improve prediction accuracy (van Binsbergen et al., 2015). On the other hand, several studies have demonstrated that the accuracy of GP could be improved by adding significant SNPs which were detected by genome‐wide association studies (GWAS) from WGS data (Brøndum et al., 2015; Warburton et al., 2020). Genetic variation affecting quantitative traits is ubiquitous in nature yet. Evolutionary force changes the allele frequency of a series of variations and results in changes in specific traits. GWAS is the most commonly used method to detect these genetic variations (Josephs et al., 2017). We speculated that the phenotypic variation of IFC in Suhuai pigs might also be affected by artificial selection, resulting in the change of allele frequency at several SNPs, which were significantly associated with IFC. A previous study found that the accuracy of genomic prediction could be improved by pruning the LD between SNPs in iWGS data (Ye et al., 2019). Moreover, various models to integrate important SNPs for GP have been used, such as one‐ or two‐component GBLUP, Bayesian mixture model and so on (Liu et al., 2020).

The accuracy of genomic selection depends on size of reference population. For the trait such as IFC, it is difficult to have a large reference population. It is unclear whether iWGS data can give an extra contribution to accuracy of genomic prediction compared with SNP chip data, in the case of a small reference population. The objectives of this study were (1) to identify significant SNPs associated with IFC and (2) to improve the accuracy of genomic prediction by integrating significant SNPs into the prediction models.

## 2 MATERIALS AND METHODS

### 2.1 Ethics approval

All experimental animals in this study were carried out in accordance with the Guidelines for the Care and Use of Laboratory Animals prepared by the Institutional Animal Welfare and Ethics Committee of Nanjing Agricultural University, Nanjing, China (Certification No: SYXK [Su] 2017–0007).

### 2.2 Pig population

A total of 482 Suhuai pigs from Huaiyin Xinhuai Pig Breeding Farm (Huaian, China) were randomly selected for this study, including 291 barrows and 191 females. These 482 pigs included 330 pigs used in our previous study (Wang et al., 2019). Briefly, these 482 Suhuai pigs mainly originated from 80 sires and 226 dams in 15 consanguinities. All growing‐finishing pigs were fed same diet and ad libitum with free access to water under standard indoor conditions. Finally, they were slaughtered at 80 ~ 90 kg (about 220 days) live weight in four batches at the same slaughterhouse from 2017 to 2019. After slaughter, *longissimus dorsi* muscle samples from the last rib of the left side carcass and ear samples were collected.

### 2.3 Phenotypic data

IFC accumulates both in (intramyocellular) and out (extramyocellular) of the muscle fibers. The IFC of 482 Suhuai pigs was measured by the Soxhlet extraction method according to a standard procedure, as described by Supakankul and Mekchay (Supakankul & Mekchay, 2016). In addition to the original phenotypes, corrected phenotypes were calculated as genomic estimated breeding values (GEBV) plus the estimated residual from a GBLUP model with 80 K SNP chip data (see the model below). The corrected phenotypes were used for subsequent GWAS and calculated the reliability of prediction in the validation population.

### 2.4 Genotype data

The genomic DNA was isolated from ear tissue using conventional phenol‐chloroform extraction method (Sambrook et al., 1982). Only high‐quality genomic DNAs from 482 samples were genotyped using GeneSeek GGP Porcine 80 K SNP chip (Neogen), which contained a total of 68,516 SNPs (Banerjee et al., 2020). The physical positions of SNPs in 80 K SNP chip were based on the *Sus scrofa* 10.2 genome of Duroc breed. To facilitate the subsequent genotype imputation, we converted the physical positions of 80 K SNP chip data to the *Sus scrofa* 11.1 by USCS website (https://genome.ucsc.edu/cgi‐bin/hgLiftOver). These 80 K chip dataset of 482 samples included 96 sample SNP data in our previous study (Wang et al., 2021). Quality control of genotype data was conducted using PLINK (v1.90 beta) (Purcell et al., 2007) to detect and exclude unreliable genotypes. The SNPs were removed if any of the following criteria were involved: (a) no chromosomal or physical location, (b) non‐autosomal variants, (c) minor allele frequency (MAF) < 0.01, or (d) call rate <0.9. In addition, the individuals missing more than 10% of genotypes were removed. After filtering, 50,482 SNPs for 482 individuals were retained in the dataset. The number of SNPs after filtering was similar to other studies (Banerjee et al., 2020).

### 2.5 Whole‐genome resequencing and variant calling

According to the pedigree data of all Suhuai pigs in the breeding farm, which contain 15 lineages, one male and one female from each lineage (a total of 30 individuals) were selected for resequencing. The resequencing was performed by the MGISEQ‐2000 sequencing platform. The details of 30 sequenced pigs are shown in Table S1. In this study, about 1.0 Tb of clean data was generated from the 30 individuals using Fastp (v0.23.1) with the command ‘‐c ‐q 20 ‐u 50 ‐n 15 ‐5 20 ‐3 20 ‐w 10’, and 978.2 Gb were mapped to the pig reference genome using BWA (v0.7.8) software with the command ‘mem ‐t 10 ‐M ‐R'. In this study, *Sus scrofa* 11.1 genome of Duroc breed was selected as the reference genome. Suhuai pig contains Chinese local pig and European pig lineage, however, compared with the reference genome of Chinese local pigs (such as Meishan pig) (Zhou et al., 2021), Luchuan pig (Yang et al., 2019), and European wild boar (Groenen et al., 2012), the reference genome of Duroc is the standard reference genome by NCBI (https://www.ncbi.nlm.nih.gov/data‐hub/genome/GCF_000003025.6/). The annotation information of the Duroc's reference genome is more complete and highly recognized, and is maintained in real time by NCBI (Warr et al., 2020). Furthermore, an important point was that the physical positions of the converted 80 K chip data were based on Duroc's reference genome, in order to improve the accuracy of subsequent imputation, the reference genome of Duroc was selected finally. Among all selected individuals, the average read mapping rates was 95.81%, the minimum value and maximum value were 94.98% and 96.55%, respectively. The possible reason was that the quality of several bases in front of each read was relatively low, which makes the mapping rate slightly low. The average uniquely mapped reads rates were 94.17% (from 93.07% to 95.87%), and the average depth of coverage was 13.30‐fold (from 10.43‐fold to 15.44‐fold). SNP calling and filtering were implemented according to the GATK Best Practices pipeline (Van der Auwera et al., 2013) and VariantFiltration options. The filter parameters were as follows: QualByDepth (QD) ≥ 2.0, Fisher‐Strand (FS) > 60.0, strand odds ratio (SOR) > 3.0, mapping qualities of reads (MQRankSum) < −12.5, ranked sum test for the distance of alleles from the end of the reads (ReadPosRankSum) < −8.0. A total of 23,765,813 SNPs were retained in the 30 Suhuai pigs. Finally, the 80 K SNP chip data and resequencing data were phased by Beagle 5.1 (Browning et al., 2018) for subsequent imputation.

### 2.6 Imputation

The imputation from the 80 K SNP chip data to WGS genotypes was performed by Beagle 5.1 (Browning et al., 2018) under the default parameter settings. During the imputing process, 30 individuals with resequencing data were used as the reference panel, and 482 individuals with 80 K SNP chip data were used as the target panel. Imputation quality control of iWGS data was based on the dosage R‐squared (DR^2^) in Beagle, which is an estimate of the squared correlation between the estimated allele dose and the true allele dose. Meanwhile, we found that some SNPs originated from 80 K data were missing after imputation, so, the genotypes of 80 K SNP chip were integrated into the iWGS data and replaced the imputed ones. To validate imputation accuracy, we calculated correlation between imputed and observed genotypes from five replicates under the condition that DR^2^ was greater than 0.9. In each replicate, 2000 SNPs in the 80 K SNP chip data of 482 individuals were randomly masked, and then imputed them. The imputed genotypes were compared with chip panel genotypes to calculate the correlation coefficient. Finally, SNPs with MAF less than 0.01 and DR^2^ less than 0.9 were removed, and 8,103,716 SNPs with high imputing accuracy were remained for subsequent GWAS.

### 2.7 Genome‐wide association study

A single marker GWAS was performed with a linear model using LDAK version 5.1 (Speed et al., 2020). The GWAS model was as follows:

(1) y=1μ+Xβ+Za+e,

where y is the vector of corrected phenotypes; **1** is a vector of ones; **μ** is the overall mean; **β** is the unknown allele substitution effect of the SNP tested for the association; **X** is the vector containing the genotype score for the tested SNP; **a** is the vector of the random polygenic effects which follow a normal distribution $a\sim N\left(0,G\sigma_{a}^{2}\right)$, where **G** is the genomic relationship matrix (GRM) which is built using 80 K or iWGS data excluding the chromosome of the SNP tested, and $\sigma_{a}^{2}$ is the additive genetic variance; **Z** is the incidence matrix for **a**, **e** is the vector of residual effects with $e\sim N\left(0,I\sigma_{e}^{2}\right)$, where **I** is an identity matrix and $\sigma_{e}^{2}$ is the residual variance. The SNP data sets used for GWAS were 80 K SNP chip data and iWGS data, respectively. The Bonferroni correction method was used to determine the threshold values of significance in this study. The genome‐wide significance level (0.05/*N*) and suggestive significance level (1/*N*) (Bland & Altman, 1995) were used in all GWAS, where *N* is the number of analyzed SNPs. Consequently, the genome‐wide significance level and suggestive significance level were *p* = 9.90E‐7 and *p* = 1.99E‐5 for 80 K SNP chip data, and the genome‐wide significance level and suggestive significance level were *p* = 6.18E‐9 and *p* = 1.24E‐7 for WGS data. In addition, we defined QTL region as an interval between significant SNPs located on the upstream and downstream of physical location. Manhattan plots were obtained using the CMplot package (Yin et al., 2021) within the R software (http://www.r‐project.org/). For the public annotation databases, clusterProfiler (Yu et al., 2012), an R package, were used to obtain Gene Ontology (GO) terms and Kyoto Encyclopedia of Genes and Genomes (KEGG) pathways in all significant regions.

The variance components of IFC were estimated and the IFC phenotype was adjusted for fixed effects (sex, batch, age and carcass weight) using the mixed linear model implemented in the DMU software (Madsen et al., 2014) based on 80 K SNP chip data, and the model was described previously (Wang et al., 2021). The phenotypic variance explained (PVE) by each locus was calculated using the equation ${\mathrm{PVE}}_{i}=\frac{2q_{i}\left(1-q_{i}\right)b_{i}^{2}}{\sigma_{p}^{2}}$, where q_i_ is the minor allele frequency of the *i*th SNP, b_i_ is the allele substitution effect of the *i*th SNP and $\sigma_{p}^{2}$ is the phenotypic variance of IFC. Heritability ($h^{2}$) of IFC was defined as the ratio of the additive genetic variance to phenotypic variance ($h^{2}=\frac{\sigma_{a}^{2}}{\sigma_{a}^{2}+\sigma_{e}^{2}}$).

### 2.8 Pre‐selection of SNPs for genomic prediction

To avoid automatically using information of validation animals to predict breeding value of the validation animal, the GWAS was performed in each generated reference population using iWGS (GWAS_iWGS_) data. Briefly, all genotyped Suhuai pigs (*n* = 482) with phenotype data were randomly divided into five separate groups, four groups were selected randomly as reference population, and the phenotype of the remaining one group was masked and used as the validation population. According to the above‐mentioned method, five reference populations were formed, and GWAS was carried out based on each reference population using Model 1, respectively. Then, the significant SNPs affecting IFC in each reference population were selected into the models to predict breeding value when using this reference population.

### 2.9 Statistical model for predicting breeding value

The statistical methods used for predicting breeding value in this study included linear mixed model using pedigree‐based relationship matrix (PBLUP), linear mixed models using genome‐wide marker‐based relationships matrix (GBLUP) and Bayesian mixture model (BayesMix). For GBLUP and BayesMix models, the genotype data sets include 80 K data, iWGS data, and pre‐selected iWGS SNPs data based on GWAS results. We assessed a one‐component model and a two‐component model on their efficiency to use the pre‐selected iWGS SNPs in these models. A one‐component model considered 80 K data and pre‐selected iWGS SNPs as one genetic component, and a two‐component model considered 80 K data and pre‐selected iWGS SNPs as two separate genetic components. It is worth noting that when one‐ or two‐component model was used for GP, the duplicate SNPs in 80 K data and pre‐selected SNPs data will be deleted from 80 K data.

In order to investigate the impact of LD‐based SNP pruning on genomic prediction using iWGS data, twenty different R‐squared cutoffs of LD (1.00, 0.95, 0.90, 0.85, 0.80, 0.75, 0.70, 0.65, 0.60, 0.55, 0.50, 0.45, 0.40, 0.35, 0.30, 0.25, 0.20, 0.15, 0.10, 0.05) were used to prune variants. LD was calculated based on the option ‐‐indep‐pairwise PLINK (v1.90 beta) in a 50‐kb sliding window with 10 variants. The number of SNPs after pruning at different cutoff levels are shown in Table S2.

### 2.10 PBLUP model

The PBLUP model is

(2) y=1μ+Xb+Zu+e,

where y is the vector of phenotype value; **1** is a vector of ones; **μ** is the overall mean; **b** is the vector of fixed effects (sex and batch as class variables, age and carcass as regression covariables); **u** is the vector of additive genetic effects, and assumed that $u\sim N\left(0,A\sigma_{u}^{2}\right)$, in which **A** is the matrix of additive genetic relationship constructed based on the pedigree; $\sigma_{u}^{2}$ is the additive genetic variance; **X** and **Z** are incidence matrices relating the fixed effects and the additive genetic values to phenotype values; **e** represents random residual effects. The prediction of breeding value with PBLUP model was performed using the HIBLUP software (https://www.hiblup.com/).

### 2.11 GBLUP model

The models for predicting breeding value using genotype information are as follows:

The one‐component GBLUP model is

(3) y=1μ+Xb+Zg+e,

The two‐component GBLUP model is

(4) $$y=1\mu +\mathrm{Xb}+Z_{1}g_{1}+Z_{1}g_{2}+e,$$

where **y**, **1**, **μ**, **X**, **b**, and **e** are the same as in Model 2; **g**, **g**_**1**_, **g**_**2**_ are vectors of additive genetic values; **Z, Z**_**1**_ **, Z**_**2**_ are the incidence matrix to assign **y** to **g, g**_**1**_ **, g**_**2**_. We assumed that the additive genetic value is $g∼N\left(0,G\sigma_{g}^{2}\right)$, $g_{1}∼N\left(0,G_{1}\sigma_{g_{1}}^{2}\right)$, and $g_{2}∼N\left(0,G_{2}\sigma_{g_{2}}^{2}\right)$, where **G** is a relationship matrix built with either 80 K, iWGS data, or 80 K together with pre‐selected SNPs; **G**_**1**_ is a relationship matrix built with 80 K data; G_2_ is a relationship matrix built with pre‐selected SNPs data; $\sigma_{g}^{2}$, $\sigma_{g_{1}}^{2}$, and $\sigma_{g_{2}}^{2}$ represent corresponding additive genetic variance, respectively. The estimated breeding value in Model 4 was defined as sum of g_1_ and g_2_. The prediction of breeding value with GBLUP model was performed using the HIBLUP software (https://www.hiblup.com/).

### 2.12 Bayesian mixture model

The one‐component BayesMix model is

(5) $$y=1\mu +\mathrm{Xb}+Z_{S}a+e,$$

The two‐component BayesMix model is

(6) $$y=1\mu +\mathrm{Xb}+Z_{S1}a_{1}+Z_{S2}a_{2}+e,$$

where **y**, **1**, **μ**, **X**, **b**, and e are the same as in Model 2; **a** is the vector of SNP effects for all SNPs in the model, **a**_**1**_, and **a**_**2**_ are the vector of effects for 80 K SNPs and the vector of effects for pre‐selected iWGS SNPs, respectively; **Z**_**s**_, **Z**_**s1**_, and **Z**_**s2**_ represent corresponding genotype matrices, respectively. For iWGS data, only pruned iWGS SNPs of scenario LD < 0.55 were used for genomic prediction using the BayesMix model. We assumed the distribution of SNP effects (**a**, **a**_**1**_, or **a**_**2**_) follows a mixture of four normal distributions:

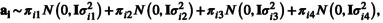

where a_*i*_ is a particular vector of SNP effects (**a**, **a**_1_ or **a**_2_); *π*_*ij*_ (*j* = 1, 2, 3 and 4) is the probability of an SNP belongs to the *j*th distribution within the *i*th component in the model, and $\sigma_{\mathrm{ij}}^{2}$ is the variance for *j*th distribution within the *i*th component. In the present study, *π*_*ij*_ is sampled from the Dirichlet distribution *π*_*ij*_ = (*π*_*i*1_, *π*_*i*2_, *π*_*i*3_, *π*_*i*4_) ~ dir (125, 25, 5, 1) with prior *π*_*i*1_ = 0.889, *π*_*i*2_ = 0.1, *π*_*i*3_ = 0.01, and *π*_*i*4_ = 0.001. $\sigma_{\mathrm{ij}}^{2}$ is assumed to have a scaled inversed $\chi^{2}$ distribution and have a fixed ratio of 1000 $\sigma_{i1}^{2}$ = 100 $\sigma_{i2}^{2}$ = 10 $\sigma_{i3}^{2}$ =  $\sigma_{i4}^{2}$; thus, only one of them is required to be estimated within each genetic component. In the present study, the BayesMix model was run using single chain Gibbs sampler with a total length of 50,000 samples, where the first 10,000 samples were discarded as burn‐in. The analyses with BayesMix models were carried out using the Bayz software (http://www.bayz.biz).

In total, nine approaches were used to estimate breeding values for IFC. (1) PBLUP with Model 2 which used the pedigree‐based relationship matrix (**A**). (2) GBLUP‐80 K with Model 3 which used the 80 K SNP chip data to calculate the GRM (**G**). (3) GBLUP‐iWGS with Model 3 which used the iWGS data pruned at different LD levels to calculate the GRM (**G**) separately. (4) GBLUP‐80 K‐GWAS_iWGS_‐one with Model 3 which pooled the pre‐selected iWGS SNPs and the 80 K SNPs together as one component to construct the GRM (**G**). (5) GBLUP‐80 K‐GWAS_iWGS_‐two with Model 4 which used the pre‐selected iWGS SNPs to construct **G**_**2**_ and the 80 K SNPs to construct **G**_**1**_; (6) Bayesian‐80 K with Model 5 which used the 80 K SNP chip data only; (7) Bayesian‐iWGS with Model 5 which used the pruned iWGS data (LD < 0.55) only. (8) Bayesian‐80 K‐GWAS_iWGS_‐one with Model 6 which use the 80 K SNPs and pre‐selected iWGS SNPs as one genetic component; (9) Bayesian‐80 K‐GWAS_iWGS_‐two with Model 6 which used the 80 K SNPs and the pre‐selected iWGS SNPs as two separate genetic components.

### 2.13 Validation of prediction

Accuracy of genomic prediction were assessed using a 5‐fold cross validation procedure. The predictability for estimating breeding values were assessed by reliability. The reliability of the prediction was measured as squared correlation between estimated breeding values and corrected phenotypes in the validation groups divided by the *h* ^2^ of IFC. The standard error (SE) was calculated as the standard deviation of the 5 calculated reliability from the 5 fold cross‐validation divided by the square root of 5.

## 3 RESULTS

### 3.1 Heritability of IFC and accuracy of genotype imputation

As shown in Table 1, the mean and standard error of IFC in 482 Suhuai pigs was 1.91 ± 0.03%, and the coefficient of variation was 31.73%, indicating a large variation of IFC phenotypes in this population. The heritability (*h* ^2^) of IFC was 0.26, estimated using a model 1. Heritability estimated from other models was also around this size.

**TABLE 1:** Descriptive statistics of intramuscular fat content (IFC)

Trait	Mean ± SE	MAX	MIN	CV	$\sigma_{a}^{2}$	$\sigma_{e}^{2}$	$h^{2}$
IFC	1.91 ± 0.03%	4.08%	0.82%	31.73%	0.07	0.21	0.26(0.08)

Abbreviations: $\sigma_{a}^{2}$, additive genetic variance; $\sigma_{e}^{2}$, residual variance; CV, coefficient of variation; *h* ^2^, heritability; IFC, intramuscular fat content; MAX, maximum value; MIN, minimum value; SE, standard error.

The number of SNPs after imputation and imputation accuracy for each autosome are shown in Table 2. For iWGS genotype data, after removing the SNPs with DR^2^ < 0.9, the corresponding values of average imputation accuracy (DR^2^) was 0.96, and 8 103,716 SNPs were remained for subsequent GWAS. The average correlation between imputed and observed genotypes was 0.93 (0.86–0.96) in the validation, which was lower than DR^2^.

**TABLE 2:** The number of SNPs after imputation and imputation accuracy in each autosome

SSC	Number of SNP		Imputation accuracy		
	Before filtering	After filtering	Before filtering (DR^2^)	After filtering (DR^2^)	After filtering (correlation)
1	2,192,379	776,910	0.68	0.96	0.94
2	1,622,820	544,049	0.70	0.96	0.90
3	1,458,780	393,133	0.66	0.95	0.92
4	1,361,417	473,520	0.70	0.96	0.89
5	1,205,203	407,256	0.69	0.96	0.92
6	1,715,624	667,613	0.73	0.96	0.94
7	1,385,906	489,889	0.73	0.96	0.94
8	1,552,180	499,215	0.69	0.96	0.92
9	1,513,650	625,566	0.74	0.96	0.92
10	1,060,758	337,762	0.71	0.95	0.95
11	938,196	274,700	0.67	0.96	0.96
12	817,647	263,914	0.69	0.95	0.94
13	1,762,651	612,196	0.69	0.96	0.93
14	1,494,414	520,096	0.71	0.96	0.95
15	1,177,386	431,954	0.67	0.96	0.94
16	1,023,279	286,951	0.70	0.95	0.95
17	828,825	263,145	0.69	0.95	0.86
18	654,698	235,847	0.70	0.96	0.92
Overall	23,765,813	8,103,716	0.70	0.96	0.93

Abbreviations: DR^2^, dosage R‐squared; SNP, single‐nucleotide polymorphisms; SSC, *Sus scrofa* chromosome.

### 3.2 Genome‐wide association study for IFC in 482 Suhuai pigs

**FIGURE 1:**
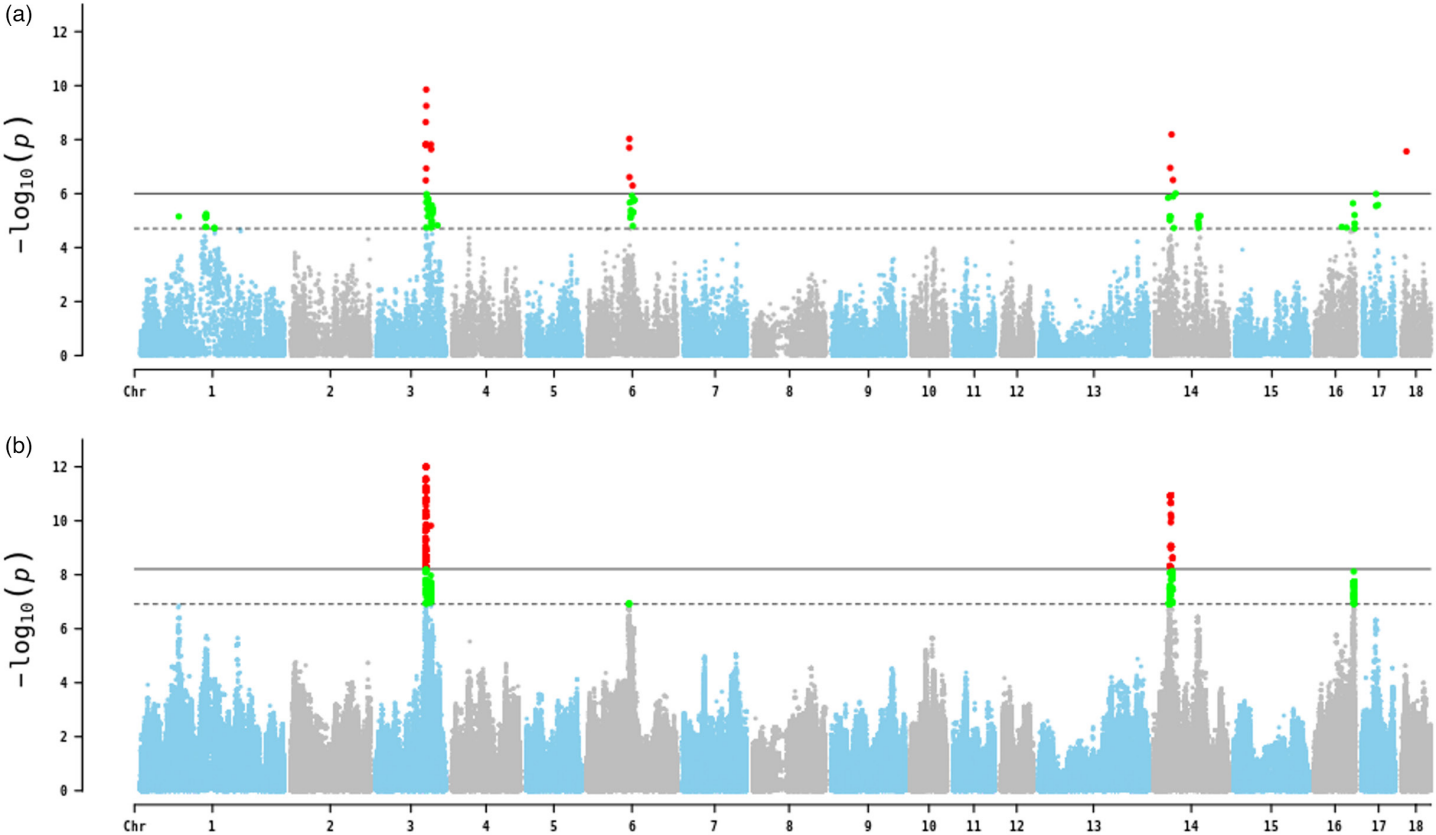
Manhattan plots of the GWAS based on 80 K data (a) and iWGS data (b). Different colors denote on different *sus scrofa* chromosomes (SSCs). The solid and dashed lines indicate the Bonferroni‐corrected thresholds of the genome‐wide and suggestive significances, respectively. The red dot represents single nucleotide polymorphism (SNP) exceed genome‐wide significance level, and the bright green dot represents SNP exceed suggestive significance level.

This study conducted GWAS in two scenarios. In the first scenario, GWAS was conducted using the 80 K data. We identified 7 QTL regions including 80 SNPs which surpassed the suggestive significance level (*p* < 1.98E‐05) and 20 SNPs which reached the genome‐wide significant level (*p* < 9.90E‐07) (Figure 1a, Table 3). These significant SNPs (suggestive or genome‐wide significance) were mainly distributed on *Sus scrofa* chromosome (SSC) 1 (124.19–125.56 Mb), SSC3 (94.40–104.56 Mb), SSC6 (79.75–89.16 Mb), SSC14 (25.80–40.17 Mb) and SSC16 (72.97–76.25 Mb). The most significant SNP ASGA0015463 (*p* = 1.41E‐10) was located near 95.27 Mb of SSC3, which could explain 2.19% of the phenotypic variance. There were three significant SNPs on SSC17 and one on SSC18, respectively, and the top SNP WU_10.2_18_10342488 on SSC18 could explain 2.80% of the phenotypic variation. In the second scenario with iWGS data, 4 QTL regions were identified. A total of 1217 suggestive‐significant SNPs were identified (*p* < 1.23659E‐07), while 517 SNPs achieved a genome‐wide significant level (*p* < 6.18296E‐09) in these QTL regions (Figure 1b, Table 3). These significant SNPs were also mainly distributed on SSC3 (94.32–105.54 Mb), SSC6 (79.75–89.16 Mb), SSC14 (29.21–35.16 Mb) and SSC16 (75.66–76.27 Mb). Compared to the first scenario, GWAS based on iWGS dataset could reduce the range of significant regions and increase the number of significant SNPs in these significant regions, especially on SSC14 and SSC16. The number of QTL regions was fewer using the iWGS than using 80 K data because significant level was at much smaller *p* value (after multiple testing correction) when using iWGS data.

**TABLE 3:** The GWAS results for IFC based on the 80 K data and iWGS data

SNP set	SSC	Range of SNP (MB)	Top SNP ID	Top SNP position (bp)	*p* value of top SNP	PVE of top SNP (%)	Number of SNP (*p* < 0.05/*N*)	Number of SNP (*p* < 1/*N*)
80 K	1	124.19–125.56	ALGA0005858	125,114,214	5.65E‐06	1.28	0	16
	3	94.40–104.56	ASGA0015463	95,269,660	1.41E‐10	2.19	11	30
	6	79.75–89.16	ALGA0035657	79,830,116	9.34E‐09	1.96	4	8
	14	25.80–40.17	ASGA0062592	32,492,848	6.47E‐09	2.14	4	15
	16	72.97–76.25	WU_10.2_16_78907155	72,978,738	2.29E‐06	1.23	0	7
	17	26.27	WU_10.2_17_29814432	26,269,281	1.03E‐06	1.58	0	3
	18	9.84	WU_10.2_18_10342488	9,835,091	2.74E‐08	2.80	1	1
iWGS	3	94.32–105.54	rs338155853	95,237,333	1.01E‐12	2.74	453	931
	6	79.82–79.84	rs344478115	79,829,725	1.19E‐07	1.62	0	4
	14	29.21–35.16	rs331439214	31,793,530	1.16E‐11	2.73	64	208
	16	75.66–76.27	rs328332885	75,669,819	7.56E‐09	2.21	0	74

Abbreviations: iWGS, imputed whole‐genome sequencing; PVE, phenotypic variance explained; SNP, single‐nucleotide polymorphisms; SSC, *Sus scrofa* chromosome.

We performed functional enrichment analysis in all significant regions, a total of 160 genes were annotated. Functional analysis of these genes revealed that there were 99 GO terms and 20 KEGG pathways significantly enriched (false discovery rates <0.05) based on *Sus scrofa* 11.1 genome (Tables S4 and S5). Moreover, through gene function analysis, protein kinase C epsilon (*PRKCE*) and myosin light chain 2 (*MYL2*) gene were suggested to be two important candidate genes affecting IFC. It is worth noting that, a total of 355 significant SNPs were located in *PRKCE* gene on SSC3, and the most significant locus rs338155853 was located near 0.33 Mb downstream of *PRKCE* gene. Moreover, the most significant SNP rs331439214 on SSC14 was located near 0.36 Mb upstream of *MYL2* gene.

### 3.3 Pre‐selection of SNPs based on GWAS_iWGS_ in five reference populations

We performed GWAS using iWGS data in the generated five reference populations, respectively. The summary of significant SNPs is shown in Table S3. Similar to the above GWAS results based on the whole data of 482 animals, these significant SNPs were also mainly distributed on SSC3 (94.32–105.54 Mb) and SSC14 (31.79–32.59 Mb). The most significant SNPs on SSC3 were near 95.00 Mb, while rs331439214 on SSC14 31,793,530 bp was always the most significant SNP associating with IFC in the five reference populations. The number of significant SNPs in GWAS_iWGS_ results based on different reference populations were different. Finally, based on five GWAS_iWGS_ results, 1292, 1128, 596, 153, and 595 suggestive significant SNPs were pre‐selected for subsequent genomic prediction in the 5‐fold cross‐validation procedure.

### 3.4 Genomic prediction

**FIGURE 2:**
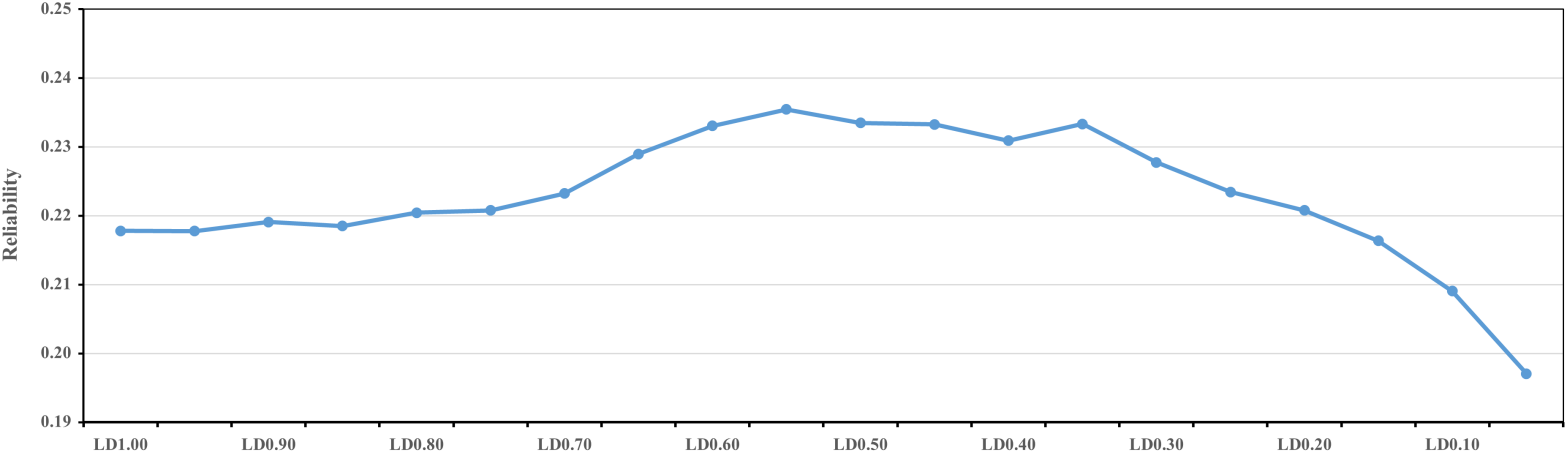
The reliability of prediction using GBLUP‐iWGS models with different R‐squared cutoffs of linkage disequilibrium to prune iWGS data.

**FIGURE 3:**
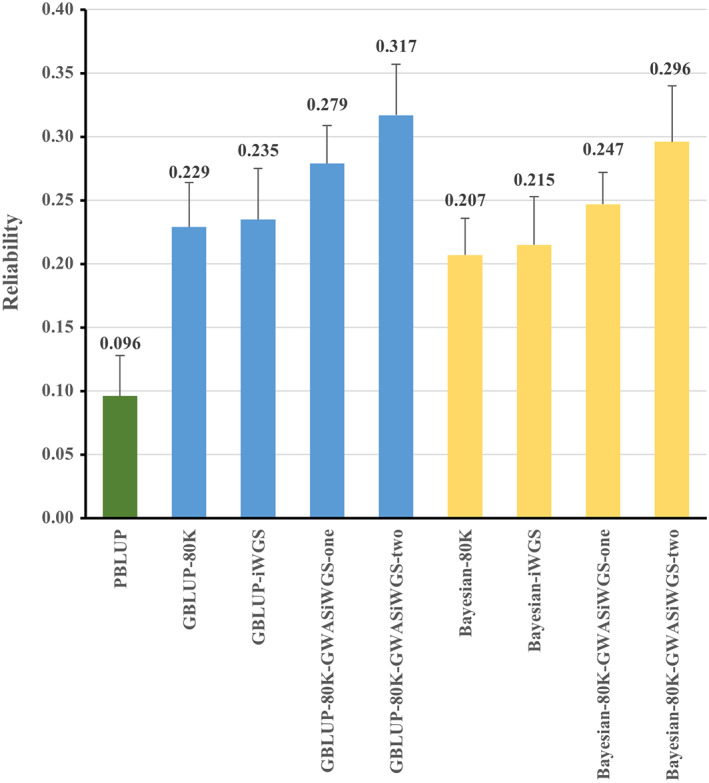
The reliability of predictions using PBLUP, GBLUP and Bayesian mixture models.

The reliabilities of prediction using PBLUP, GBLUP and BayesMix in validation populations are presented in Figures 2 and 3. In this study, the PBLUP model had the lowest prediction reliability (0.096 ± 0.032). The reliability (0.229 ± 0.035) of genome prediction was significantly improved by replacing pedigree information with 80 K SNP chip data. Compared with 80 K SNP chip data in GBLUP model, the reliability of prediction was reduced by 0.011 when whole iWGS SNPs were used. When using pruned iWGS SNPs, pruning using the R‐squared cutoff of LD at 0.40 to 0.60 led to higher accuracies with the highest accuracy for cutoff of LD at 0.55, which led to a slight improvement (0.006) compared with GBLUP‐80 K. For GBLUP models, compared with using the 80 K SNPs alone, adding additional pre‐selected iWGS SNPs to the 80 K SNPs and treating all the SNPs as one genetic component (GBLUP‐80 K‐GWAS_iWGS_‐one) led to a significant improvement of reliability by 0.05. Moreover, the GBLUP model treating the two sets of SNPs as two genetic components (GBLUP‐80 K‐GWAS_iWGS_‐two) resulted in an increase of reliability which was 0.033 higher than that of the one‐component GBLUP model. For BayesMix models, compared with using 80 K SNPs alone, adding additional pre‐selected iWGS SNPs to the 80 K SNPs also led to significant improvements of reliabilities, and the reliability of prediction were improved by 0.040 when using one‐component model and 0.089 when using two‐component model, respectively. Similar to results of GBLUP model, compared with using 80 K SNPs, Bayesian‐iWGS (LD < 0.55) model led to slight improvement (0.008) of reliability. In this study, the prediction reliabilities of GBLUP models were better than those of BayesMix models when using the same SNPs dataset.

## 4 DISCUSSION

### 4.1 Genome‐wide association study

In the present study, GWAS based on 80 K SNP chip data and iWGS data were carried out to explore key variants affecting IFC in 482 Suhuai pigs. The consistency of these two GWAS results was relatively high, and the identified significant regions affecting IFC mainly distributed on SSC3, SSC6, SSC14 and SSC16. In this study, the use of iWGS data instead of 80 K data improved the detection power of SNPs of interest, association analyses using iWGS data rather than 80 K data could highlight the peaks and increase the phenotypic variation explained by the peak SNP in each QTL. Besides, GWAS using iWGS data rather than 80 K data could reduce the mapping noise. Several significant SNPs on SSC1, SSC17 and SSC18 were detected based on 80 K dataset, however, the *p* values of these SNPs were larger than those in GWAS based on iWGS data. One possible reason was that the genomic relationship matrices in the models were constructed with different SNP data sets. The higher *p* values could be also resulted from larger sample size, in our previous studies, a QTL on SSC5 affecting IFC was identified by using GWAS based on 80 K dataset in high (*n* = 48) and low (*n* = 48) IFC groups (Wang et al., 2021), and the rs1110770079 SNP located on *FABP3* was significantly associated with IFC by association analysis in 330 Suhuai pigs (Wang et al., 2019). However, this significant signal on SSC5 and rs1110770079 SNP were not detected in current study, we speculated that the significant signals of early research were detected based on a limited sample size, and the phenotypic variations explained were small. When we used a larger sample size and iWGS data, four novel QTLs that could explain larger phenotypic variations were detected, and the signal peaks in our previous studies were disappeared. Therefore, it was essential to increase the sample size and the density of SNPs (e.g. iWGS data) in GWAS. Meanwhile, these significant signals with large effects derived from iWGS data might be beneficial to improve the accuracy of genomic prediction for IFC.

In the results of GWAS based on iWGS, a total of 932 SNPs significantly associated with IFC were identified in the 94.33–104.56 Mb on SSC3, of which 355 significant SNPs were located in protein kinase C epsilon (*PRKCE*) gene. *PRKCE* gene is a member of PKC family. In many studies involving cultured cells, mouse models and humans, the *PRKCE* was found being associated with the generation of lipid‐induced insulin resistance in peripheral tissues, and *PRKCE* was one of the most often implicated isoforms in PKC family (Schmitz‐Peiffer & Biden, 2008). A previous study has concluded that liver triglycerides and diacylglycerols increased in fat‐fed *PRKCE* knockout mice at 1 week, which was due to the changes in lipid distribution and promoting the esterification of fatty acids in hepatocytes (Raddatz et al., 2011). However, for the mice with *PRKCE* ^−/−^, the hepatic lipid accumulation was equivalently increased at 16 weeks, indicating that the effect of *PRKCE* gene on lipid metabolism was time‐dependent (Raddatz et al., 2011). Puig‐Oliveras et al. performed an expression‐based GWAS in swine muscle (*Longissimus dorsi*) and found that *PRKCE* gene was contained in a *trans*‐eQTL for Acyl‐CoA Synthetase Medium‐Chain Family Member 5 (ACSM5) (Puig‐Oliveras et al., 2016), and ACSM5 was proved to play an important role in activating first step fatty acid metabolism (Gyamfi et al., 2021). Therefore, we speculated that *PRKCE* gene was a key candidate gene affecting fat metabolism. Further study is needed to investigate its function on IFC deposition in pigs.

In the second significant region based on iWGS data, SSC14 (29.21–35.16 Mb), a total of 208 significant SNPs were identified. The most significant SNP rs331439214 on this region was located near 0.36 Mb upstream of myosin might chain 2 (*MYL2*) gene. *MYL2* is a major sarcomeric protein in mammalian striated muscle, and plays a pivotal role in the development and function of the heart (Sheikh et al., 2015). *MYL2*, *MYL3*, *MYH7* and *TPM3* were considered to be the four best hits coded for typical slow muscle proteins (Amann et al., 2014), previous research found that slow fibers exhibit a higher intramyocellular lipid content than fast glycolytic fibers (Essén‐Gustavsson et al., 1994). Pan et al. (2020) performed transcriptome sequencing in *longissimus dorsi* muscle tissue of Luchuan pig and Duroc pig and found forty differentially expressed genes (DEGs) related to muscle development, including *MYL2*. Given that *MYL2* might affect fatty acid metabolism, we speculated that it might also be considered as a candidate gene for IFC in pigs.

### 4.2 Genomic prediction

This study applied PBLUP, GBLUP and Bayesian four‐distribution mixture model with different sets of marker genotypes, for genetic evaluation of IFC in Suhuai pigs. The predictive abilities of these three methods were compared in terms of prediction reliability. Our results indicated that the methods based on marker information were more accurate than the method based on pedigree alone. Similarly, Uemoto et al. (2017) reported that the benefits of genomic prediction in terms of increased accuracy of prediction by about 21% over a pedigree‐based model for IFC in a closed line of Duroc pigs. This is likely due to the fact that the GBLUP and Bayesian methods have a better capture of the Mendelian sampling terms in comparison with the pedigree‐based prediction method (Chen et al., 2015).

Using WGS data in genomic prediction is expected to bring higher predictive reliability, because WGS data included causal SNPs affecting target traits (Meuwissen & Goddard, 2010; Song et al., 2019). Meuwissen and Goddard (2010) demonstrated in simulations that genomic predictions based on WGS data were 5%–10% more accurate than predictions based even on high dense markers, because the causal mutations were used in prediction. However, in the current study, the prediction reliability of model with GRM constructed using whole iWGS data were slightly lower than using 80 K data. Similar to our results, previous studies on reproduction and production traits in pig (Song et al., 2019; Van Binsbergen et al., 2015), reproduction and conformation trait in cattle (Frischknecht et al., 2018), carcass and meat quality in sheep (Moghaddar et al., 2019) suggested no improvement or only marginal increase in genomic prediction accuracy when using of iWGS, compared with chip data. Several factors could explain the unsatisfied results of genomic prediction using iWGS data. First, in iWGS data, a larger number of SNPs do not link to the QTLs affecting the traits of interest, thus, more noise might be introduced to prediction. Second, in the current study, thirty key individuals based on pedigree relationship were selected as the reference panel for resequencing, and the imputation from clean 80 K data to WGS data was performed. However, potential imputation errors were difficult to detect and eliminate, which also affected the accuracy of genomic prediction. Calus et al. (2014) have reported that low imputation accuracy had a negative impact on genomic prediction accuracy, thereby reducing the response to selection.

Several studies on genomic prediction for multiple traits often pruned WGS data with fixed LD value (Brøndum et al., 2015; Song et al., 2019). In this study, we investigated the impact of pruning at different levels on genomic prediction. The results showed that moderate LD (LD < 0.55) resulted in higher prediction reliability when using all iWGS data. However, Ye et al. (2019) found that the prediction accuracies of most traits using iWGS data were the highest when lower LD (0.10) was used to prune markers. We speculated that an important reason could be LD degree in different population. The number of remaining SNPs was large in low LD population, and small in high LD population under the same cutoff threshold. Another possible explanation is due to the difference in genetic structure of traits.

Many studies have demonstrated that adding significant SNPs derived from high‐density marker panels or WGS data into medium density panel data could improve the accuracy of GP (Brøndum et al., 2015; Lopes et al., 2017). Brøndum et al. (2015) found that the prediction reliability of production traits in cattle could be improved by 3 to 5 percentage points by including markers significant from GWAS based on WGS data alongside the 54 K SNP data sets. Similarly, in current study the significant SNPs were preselected from GWAS_iWGS_ and added to the 80 K SNPs in GBLUP models and Bayesian models, the predicted reliabilities were significantly increased. We speculated that the pre‐selected markers obtained from iWGS data in our study might be closer to the causal mutations for IFC.

To select a model to use the information of selected iWGS SNPs efficiently, we compared GBLUP models with BayesMix models by polling the selected SNP with the 80 K data or taking them as an independent component. Bayesian method is expected to perform better than GBLUP for traits controlled by loci with large effects (Lee et al., 2017), otherwise, GBLUP performs as well as Bayesian models. In this study, the prediction reliabilities of GBLUP models for IFC were better than that of Bayesian four‐distribution model when using the same SNPs dataset. Similar to our results, Chen et al. (2015) reported that the GBLUP models outperformed Bayesian for carcass marbling score trait in 543 Angus and 400 Charolais beef cattle. A possible reason may be due to the IFC trait was controlled by multiple SNPs with small effects. Another reason could be that the reference population in the current study is small and the Bayesian model is not sufficient to distinguish the SNPs with large or small effects appropriately. Karaman et al. (2016) reported that Bayesian variable selection models have no advantage over GBLUP when the size of reference population was small.

In a conventional GBLUP model, an important assumption is that all SNPs (whether or not associated with specific traits or not) have the same variance and follow the same normal distribution. However, the assumption may be not appropriate since some SNPs have large effect on the target traits than the others. In contrast, previous studies reported that a method called weighted GBLUP (WGBLUP) which put unequal weights on different SNPs led to higher accuracies than the unweighted counterpart (Su et al., 2014; Zhang et al., 2010). The WGBLUP model has similar computation costs as the regular GBLUP model but is able to reach similar reliabilities as the Bayesian mixture model. Similar to the WGBLUP concept (Zhang et al., 2010), a two‐component GBLUP model including an additional G matrix for the highly informative SNPs can differentiate the highly informative SNPs from the other SNPs. In this study, the two‐component GBLUP model (GBLUP‐80 K‐GWAS_iWGS_‐two) achieved higher reliabilities than the one component GBLUP model. Liu et al. (2020) also reported that the use of selected WGS SNPs together with the 54 K SNP chip in a two‐component GBLUP model increased the prediction reliability for milk production traits in dairy cattle. Similar to the GBLUP model, the advantage of two component approach was observed when using the Bayesian mixture model in the current study, though the Bayesian model is able to differentiate SNPs with large or small effects. The reason could be that dividing the SNPs into two sets provides good priors to the model, which could be important, especially for small reference data.

In conclusion, we implemented GWAS using iWGS data and identified two candidate genes (*PRKCE* and *MYL2*) and a number of significant SNPs for IFC, providing useful knowledge for further identification of the causal genes affecting IFC deposition. The validation of prediction showed that the models with genomic marker information could have higher prediction abilities than the methods based on pedigree alone. Compared with genomic prediction only using the chip genotype data, using all iWGS SNPs led to lower accuracy, but adding important SNPs selected from GWAS based on iWGS data to the 80 K SNPs increased accuracy. When including the important iWGS SNPs, the two‐components models performed better that the one‐component models. In addition, the 80 K chip data was imputed into WGS data based on LD, which increased the SNP density, and the causal variants affecting IFC could be more accurately identified through GWAS based on the iWGS dataset. This provides opportunity to improve pork quality by gene editing technology in the future.

## CONFLICT OF INTEREST

The authors declare that they have no conflict of interest.

## Supporting information


**Appendix S1** Supporting Information

## Data Availability

The WGS data for this study can be found in the NCBI Sequence Read Archive (SRA) under Bioproject: PRJNA791712. The 80K SNP chip data and phenotype data for 482 Suhuai pigs used in this study were deposited at the figshare repository (https://doi.org/10.6084/m9.figshare.19129349).

## References

[eva13496-bib-0001] Amann, R. , Wyder, S. , Slavotinek, A. M. , & Trueb, B. (2014). The FgfrL1 receptor is required for development of slow muscle fibers. Developmental Biology, 394(2), 228–241. 10.1016/j.ydbio.2014.08.016 25172430

[eva13496-bib-0002] Banerjee, P. , Carmelo, V. A. O. , & Kadarmideen, H. N. (2020). Genome‐wide epistatic interaction networks affecting feed efficiency in Duroc and landrace pigs. Frontiers in Genetics, 11, 121–135. 10.3389/fgene.2020.00121 32184802 PMC7058701

[eva13496-bib-0003] Bland, J. M. , & Altman, D. G. (1995). Multiple significance tests: The Bonferroni method. British Medical Journal, 310(6973), 170. 10.1136/bmj.310.6973.170 7833759 PMC2548561

[eva13496-bib-0004] Brøndum, R. F. , Su, G. , Janss, L. , Sahana, G. , Guldbrandtsen, B. , Boichard, D. , & Lund, M. S. (2015). Quantitative trait loci markers derived from whole genome sequence data increases the reliability of genomic prediction. Journal of Dairy Science, 98(6), 4107–4116. 10.3168/jds.2014-9005 25892697

[eva13496-bib-0005] Browning, B. L. , Zhou, Y. , & Browning, S. R. (2018). A one‐penny imputed genome from next‐generation reference panels. The American Journal of Human Genetics, 103(3), 338–348. 10.1016/j.ajhg.2018.07.015 30100085 PMC6128308

[eva13496-bib-0006] Calus, M. P. L. , Bouwman, A. C. , Hickey, J. M. , Veerkamp, R. F. , & Mulder, H. A. (2014). Evaluation of measures of correctness of genotype imputation in the context of genomic prediction: A review of livestock applications. Animal, 8(11), 1743–1753. 10.1017/S1751731114001803 25045914

[eva13496-bib-0007] Chen, L. , Vinsky, M. , & Li, C. (2015). Accuracy of predicting genomic breeding values for carcass merit traits in Angus and Charolais beef cattle. Animal Genetics, 46(1), 55–59. 10.1111/age.12238 25393962

[eva13496-bib-0008] Cho, I. C. , Yoo, C. K. , Lee, J. B. , Jung, E. J. , Han, S. H. , Lee, S. S. , Ko, M. S. , Lim, H. T. , & Park, H.‐B. (2015). Genome‐wide QTL analysis of meat quality‐related traits in a large F2 intercross between landrace and Korean native pigs. Genetics Selection Evolution, 47(1), 7–14. 10.1186/s12711-014-0080-6 PMC4336478 25888076

[eva13496-bib-0009] Essén‐Gustavsson, B. , Karlsson, A. , Lundström, K. , & Enfält, A.‐C. (1994). Intramuscular fat and muscle fibre lipid contents in halothane‐gene‐free pigs fed high or low protein diets and its relation to meat quality. Meat Science, 38(2), 269–277. 10.1016/0309-1740(94)90116-3 22059664

[eva13496-bib-0010] Frischknecht, M. , Meuwissen, T. H. E. , Bapst, B. , Seefried, F. R. , Flury, C. , Garrick, D. , Signer‐Hasler, H. , Stricker, C. , Bieber, A. , Fries, R. , Russ, I. , Sölkner, J. , Bagnato, A. , & Gredler‐Grandl, B. (2018). Short communication: Genomic prediction using imputed whole‐genome sequence variants in Brown swiss cattle. Journal of Dairy Science, 101(2), 1292–1296. 10.3168/jds.2017-12890 29153527

[eva13496-bib-0011] Groenen, M. A. M. , Archibald, A. L. , Uenishi, H. , Tuggle, C. K. , Takeuchi, Y. , Rothschild, M. F. , Rogel‐Gaillard, C. , Park, C. , Milan, D. , Megens, H.‐J. , Li, S. , Larkin, D. M. , Kim, H. , Frantz, L. A. F. , Caccamo, M. , Ahn, H. , Aken, B. L. , Anselmo, A. , Anthon, C. , … Schook, L. B. (2012). Analyses of pig genomes provide insight into porcine demography and evolution. Nature, 491(7424), 393–398. 10.1038/nature11622 23151582 PMC3566564

[eva13496-bib-0012] Gyamfi, J. , Yeo, J. H. , Kwon, D. , Min, B. S. , Cha, Y. J. , Koo, J. S. , Jeong, J. , Lee, J. , & Choi, J. (2021). Interaction between CD36 and FABP4 modulates adipocyte‐induced fatty acid import and metabolism in breast cancer. NPJ Breast Cancer, 7(1), 1–18. 10.1038/s41523-021-00324-7 34561446 PMC8463699

[eva13496-bib-0013] Jia, Y. , & Jannink, J.‐L. (2012). Multiple‐trait genomic selection methods increase genetic value prediction accuracy. Genetics, 192(4), 1513–1522. 10.1534/genetics.112.144246 23086217 PMC3512156

[eva13496-bib-0014] Josephs, E. B. , Stinchcombe, J. R. , & Wright, S. I. (2017). What can genome‐wide association studies tell us about the evolutionary forces maintaining genetic variation for quantitative traits? New Phytologist, 214(1), 21–33. 10.1111/nph.14410 28211582

[eva13496-bib-0015] Karaman, E. , Cheng, H. , Firat, M. Z. , Garrick, D. J. , & Fernando, R. L. (2016). An upper bound for accuracy of prediction using GBLUP. PLoS One, 11(8), e0161054. 27529480 10.1371/journal.pone.0161054 PMC4986954

[eva13496-bib-0016] Larmer, S. G. , Sargolzaei, M. , Brito, L. F. , Ventura, R. V. , & Schenkel, F. S. (2017). Novel methods for genotype imputation to whole‐genome sequence and a simple linear model to predict imputation accuracy. BMC Genetics, 18(1), 1–12. 10.1186/s12863-017-0588-1 29281958 PMC5746022

[eva13496-bib-0017] Lee, J. , Cheng, H. , Garrick, D. , Golden, B. , Dekkers, J. , Park, K. , Lee, D. , & Fernando, R. (2017). Comparison of alternative approaches to single‐trait genomic prediction using genotyped and non‐genotyped Hanwoo beef cattle. Genetics Selection Evolution, 49(1), 1–9. 10.1186/s12711-016-0279-9 PMC5240330 28093065

[eva13496-bib-0018] Liu, A. , Lund, M. S. , Boichard, D. , Karaman, E. , Fritz, S. , Aamand, G. P. , Nielsen, U. S. , Wang, Y. , & Su, G. (2020). Improvement of genomic prediction by integrating additional single nucleotide polymorphisms selected from imputed whole genome sequencing data. Heredity, 124(1), 37–49. 10.1038/s41437-019-0246-7 31278370 PMC6906477

[eva13496-bib-0019] Lopes, M. S. , Bovenhuis, H. , van Son, M. , Nordbø, Ø. , Grindflek, E. H. , Knol, E. F. , & Bastiaansen, J. W. M. (2017). Using markers with large effect in genetic and genomic predictions. Journal of Animal Science, 95(1), 59–71. 10.2527/jas2016.0754 28177367

[eva13496-bib-0020] Madsen, P. , Jensen, J. , Labouriau, R. , Christensen, O.F. , & Sahana, G. (2014). DMU‐a package for analyzing multivariate mixed models in quantitative genetics and genomics. Pages 18–22 in proceedings of the 10th world congress of genetics applied to livestock production.

[eva13496-bib-0021] Meuwissen, T. , & Goddard, M. (2010). Accurate prediction of genetic values for complex traits by whole‐genome resequencing. Genetics, 185(2), 623–631. 10.1093/genetics/185.2.NP 20308278 PMC2881142

[eva13496-bib-0022] Moghaddar, N. , Khansefid, M. , van der Werf, J. H. J. , Bolormaa, S. , Duijvesteijn, N. , Clark, S. A. , Swan, A. A. , Daetwyler, H. D. , & MacLeod, I. M. (2019). Genomic prediction based on selected variants from imputed whole‐genome sequence data in Australian sheep populations. Genetics Selection Evolution, 51(1), 1–14. 10.1186/s12711-019-0514-2 PMC6896509 31805849

[eva13496-bib-0023] Ngapo, T. M. (2017). Consumer preferences for pork chops in five Canadian provinces. Meat Science, 129, 102–110. 10.1016/j.meatsci.2017.02.022 28268205

[eva13496-bib-0024] Pan, P. , Qin, Z. , Xie, W. , Jiao, D. , Chen, B. , Guan, Z. , & Xie, B. (2020). Identification of differentially expressed genes in the longissimus dorsi muscle of Luchuan and Duroc pigs by transcriptome sequencing. *bioRxiv* . 10.1101/2020.07.01.182519 PMC9859529 36672873

[eva13496-bib-0025] Puig‐Oliveras, A. , Revilla, M. , Castelló, A. , Fernández, A. I. , Folch, J. M. , & Ballester, M. (2016). Expression‐based GWAS identifies variants, gene interactions and key regulators affecting intramuscular fatty acid content and composition in porcine meat. Scientific Reports, 6(1), 1–12. 10.1038/srep31803 27666082 PMC4989154

[eva13496-bib-0026] Purcell, S. , Neale, B. , Todd‐Brown, K. , Thomas, L. , Ferreira, M. A. R. , Bender, D. , Maller, J. , Sklar, P. , de Bakker, P. I. W. , Daly, M. J. , & Sham, P. C. (2007). PLINK: A tool set for whole‐genome association and population‐based linkage analyses. American Journal of Human Genetics, 81(3), 559–575. 10.1086/519795 17701901 PMC1950838

[eva13496-bib-0027] Raddatz, K. , Turner, N. , Frangioudakis, G. , Liao, B. M. , Pedersen, D. J. , Cantley, J. , Wilks, D. , Preston, E. , Hegarty, B. D. , Leitges, M. , Raftery, M. J. , Biden, T. J. , & Schmitz‐Peiffer, C. (2011). Time‐dependent effects of Prkce deletion on glucose homeostasis and hepatic lipid metabolism on dietary lipid oversupply in mice. Diabetologia, 54(6), 1447–1456. 10.1007/s00125-011-2073-0 21347625

[eva13496-bib-0028] Salek Ardestani, S. , Jafarikia, M. , Sargolzaei, M. , Sullivan, B. , & Miar, Y. (2021). Genomic prediction of average daily gain, Back‐fat thickness, and loin muscle depth using different genomic tools in Canadian swine populations. Frontiers in Genetics, 12(6), 1–13. 10.3389/fgene.2021.665344 PMC8209496 34149806

[eva13496-bib-0029] Sambrook, J. , Fritsch, F. E. , & Maniatis, T. (1982). Molecular cloning: A laboratory manual. Cold Spring Harbour Press., 33, 721–722. 10.2307/1309366

[eva13496-bib-0030] Schmitz‐Peiffer, C. , & Biden, T. J. (2008). Protein kinase C function in muscle, liver, and β‐cells and its therapeutic implications for type 2 diabetes. Diabetes, 57(7), 1774–1783. 10.2337/db07-1769 18586909 PMC2453608

[eva13496-bib-0031] Sheikh, F. , Lyon, R. C. , & Chen, J. (2015). Functions of myosin light chain‐2 (MYL2) in cardiac muscle and disease. Gene, 569(1), 14–20. 10.1016/j.gene.2015.06.027 26074085 PMC4496279

[eva13496-bib-0032] Song, H. , Ye, S. , Jiang, Y. , Zhang, Z. , Zhang, Q. , & Ding, X. (2019). Using imputation‐based whole‐genome sequencing data to improve the accuracy of genomic prediction for combined populations in pigs. Genetics Selection Evolution, 51(1), 1–13. 10.1186/s12711-019-0500-8 PMC6805481 31638889

[eva13496-bib-0033] Speed, D. , Holmes, J. , & Balding, D. J. (2020). Evaluating and improving heritability models using summary statistics. Nature Genetics, 52(4), 458–462. 10.1038/s41588-020-0600-y 32203469

[eva13496-bib-0034] Su, G. , Christensen, O. F. , Janss, L. , & Lund, M. S. (2014). Comparison of genomic predictions using genomic relationship matrices built with different weighting factors to account for locus‐specific variances. Journal of Dairy Science, 97(10), 6547–6559. 10.3168/jds.2014-8210 25129495

[eva13496-bib-0035] Supakankul, P. , & Mekchay, S. (2016). Association of NLK polymorphisms with intramuscular fat content and fatty acid composition traits in pigs. Meat Science, 118, 61–65. 10.1016/j.meatsci.2016.03.025 27050409

[eva13496-bib-0036] Uemoto, Y. , Sato, S. , Kikuchi, T. , Egawa, S. , Kohira, K. , Sakuma, H. , Miyashita, S. , Arata, S. , Kojima, T. , & Suzuki, K. (2017). Genomic evaluation using SNP‐ and haplotype‐based genomic relationship matrices in a closed line of Duroc pigs. Animal Science Journal, 88(10), 1465–1474. 10.1111/asj.12805 28557153

[eva13496-bib-0037] van Binsbergen, R. , Calus, M. P. L. , Bink, M. C. A. M. , van Eeuwijk, F. A. , Schrooten, C. , & Veerkamp, R. F. (2015). Genomic prediction using imputed whole‐genome sequence data in Holstein Friesian cattle. Genetics Selection Evolution, 47(1), 1–13. 10.1186/s12711-015-0149-x PMC4574568 26381777

[eva13496-bib-0038] Van der Auwera, G. A. , Carneiro, M. O. , Hartl, C. , Poplin, R. , del Angel, G. , Levy‐Moonshine, A. , Jordan, T. , Shakir, K. , Roazen, D. , Thibault, J. , Banks, E. , Garimella, K. V. , Altshuler, D. , Gabriel, S. , & DePristo, M. A. (2013). From FastQ data to high‐confidence variant calls: The genome analysis toolkit best practices pipeline. Current Protocols in Bioinformatics, 43(1), 10–11. 10.1002/0471250953.bi1110s43 PMC4243306 25431634

[eva13496-bib-0039] Wang, B. B. , Hou, L. M. , Zhou, W. D. , Liu, H. , Tao, W. , Wu, W. , Niu, P. P. , Zhang, Z. P. , Zhou, J. , & Li, Q. (2021). Genome‐wide association study reveals a quantitative trait locus and two candidate genes on sus scrofa chromosome 5 affecting intramuscular fat content in Suhuai pigs. Animal, 15(9), 100341. 10.1016/j.animal.2021.100341 34425484

[eva13496-bib-0040] Wang, B. B. , Li, P. H. , Zhou, W. D. , Gao, C. , Liu, H. , Li, H. , Niu, P. P. , Zhang, Z. P. , Li, Q. , Zhou, J. , & Huang, R. H. (2019). Association of Twelve Candidate Gene Polymorphisms with the intramuscular fat content and average backfat thickness of Chinese Suhuai pigs. Animals, 9(11), 858–874. 10.3390/ani9110858 31652864 PMC6912197

[eva13496-bib-0041] Warburton, C. L. , Engle, B. N. , Ross, E. M. , Costilla, R. , Moore, S. S. , Corbet, N. J. , Allen, J. M. , Laing, A. R. , Fordyce, G. , Lyons, R. E. , McGowan, M. R. , Burns, B. M. , & Hayes, B. J. (2020). Use of whole‐genome sequence data and novel genomic selection strategies to improve selection for age at puberty in tropically‐adapted beef heifers. Genetics Selection Evolution, 52(1), 1–13. 10.1186/s12711-020-00547-5 PMC7251835 32460805

[eva13496-bib-0042] Warr, A. , Affara, N. , Aken, B. , Beiki, H. , Bickhart, D. M. , Billis, K. , Chow, W. , Eory, L. , Finlayson, H. A. , & Flicek, P. (2020). An improved pig reference genome sequence to enable pig genetics and genomics research. Gigascience, 9(6), giaa051. 10.1093/gigascience/giaa051 32543654 PMC7448572

[eva13496-bib-0043] Wood, J. D. , Nute, G. R. , Richardson, R. I. , Whittington, F. M. , Southwood, O. , Plastow, G. , Mansbridge, R. , Da Costa, N. , & Chang, K. C. (2004). Effects of breed, diet and muscle on fat deposition and eating quality in pigs. Meat Science, 67(4), 651–667. 10.1016/j.meatsci.2004.01.007 22061815

[eva13496-bib-0044] Yang, Y. , Lian, J. , Xie, B. , Chen, M. , Niu, Y. , Li, Q. , Liu, Y. , Yi, G. , Fan, X. , & Tang, Y. (2019). Chromosome‐scale de novo assembly and phasing of a Chinese indigenous pig genome. *BioRxiv*, 770958. 10.1101/770958

[eva13496-bib-0045] Ye, S. P. , Gao, N. , Zheng, R. R. , Chen, Z. T. , Teng, J. , Yuan, X. , Zhang, H. , Chen, Z. , Zhang, X. , Li, J. , & Zhang, Z. (2019). Strategies for obtaining and pruning imputed whole‐genome sequence data for genomic prediction. Frontiers in Genetics, 10(6), 673. 10.3389/fgene.2019.00673 31379929 PMC6650575

[eva13496-bib-0046] Yin, L. , Zhang, H. , Tang, Z. , Xu, J. , Yin, D. , Zhang, Z. , Yuan, X. , Zhu, M. , Zhao, S. , & Li, X. (2021). Rmvp: A memory‐efficient, visualization‐enhanced, and parallel‐accelerated tool for genome‐wide association study. Genomics, Proteomics & Bioinformatics, 19(4), 619–628. 10.1016/j.gpb.2020.10.007 PMC9040015 33662620

[eva13496-bib-0047] Yu, G. C. , Wang, L. G. , Han, Y. , & He, Q. Y. (2012). clusterProfiler: An R package for comparing biological themes among gene clusters. Omics: a journal of integrative biology, 16(5), 284–287. 10.1089/omi.2011.0118 22455463 PMC3339379

[eva13496-bib-0048] Zhang, Z. , Liu, J. , Ding, X. , Bijma, P. , de Koning, D.‐J. , & Zhang, Q. (2010). Best linear unbiased prediction of genomic breeding values using a trait‐specific marker‐derived relationship matrix. PloS One, 5(9), e12648. 10.1371/journal.Pone.0012648 20844593 PMC2936569

[eva13496-bib-0049] Zhou, R. , Li, S. , Yao, W. , Xie, C. , Chen, Z. , Zeng, Z. , Wang, D. , Xu, K. , Shen, Z. , & Mu, Y. (2021). The Meishan pig genome reveals structural variation‐mediated gene expression and phenotypic divergence underlying Asian pig domestication. Molecular Ecology Resources, 21(6), 2077–2092. 10.1111/1755-0998.13396 33825319

